# Overstretch causes lipid accumulation in vascular smooth muscle cells dependent on NADPH oxidase 1

**DOI:** 10.1016/j.mbm.2025.100129

**Published:** 2025-03-26

**Authors:** Jiazhen Zhang, Qinfen Li, Suoqi Ding, Wei Xu, Jilei Su, Jingang Cui, Yongsheng Ding

**Affiliations:** a College of Life Sciences, University of Chinese Academy of Sciences, Beijing 101408, China; b School of Chemistry, Beihang University, Beijing, 100191, China

**Keywords:** Overstretch, NADPH oxidase 1, Lipid accumulation, Vascular smooth muscle cells, Atherosclerosis

## Abstract

At the bend and bifurcation of arteries prone to atherosclerosis, pulsatile blood retention may cause overstretch on the tube wall. It has been reported that more than half of the foam cells found in atherosclerotic plaques are derived from vascular smooth muscle cells (VSMCs), but the mechanism is not adequately understood. In this work, we used a microfluidic device to apply a cyclic stretch (15 ​% and 0.05 ​Hz) on the VSMC for 24 ​h. The stretch caused a significant increase in the intracellular lipid accumulation, accompanying with the increased NOX1 and CD36 protein expression. On the other hand, inhibition of NOX1 activity, elimination of reactive oxygen species (ROS), or knockdown of NOX1 expression could significantly inhibit intracellular lipid accumulation. In addition, the NOX1 upregulation caused by 15 ​% stretch was related to the JAK/STAT signaling pathway. Our results reveal a novel mechanism of VSMC foam cell formation caused by the upregulation of NOX1.

## Graphical abstract

**Figure:**
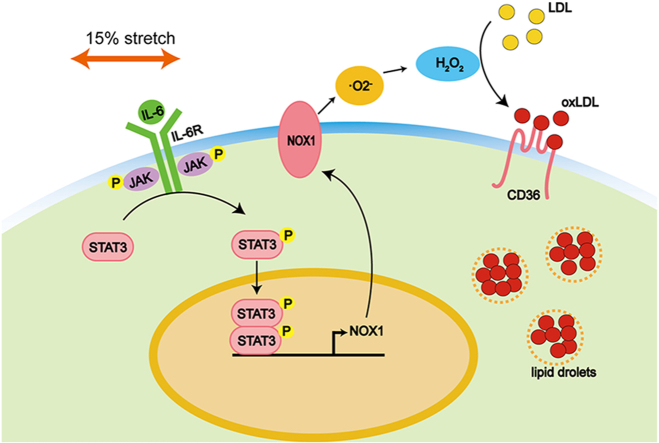

## 1 Introduction

Atherosclerosis is a chronic inflammatory disease characterized by lipid and fibrous components accumulation in arterial wall.^1^ Typically, the formation of foam cells is thought to be the result of excessive oxidized low-density lipoprotein (ox-LDL) engulfed by monocytes that infiltrate the sub-endothelial layer. However, vascular smooth muscle cells (VSMCs) undergo a transition from contractile to synthetic in response to pro-inflammatory factors around the necrotic core, promoting their migration and macrophage-like transformation.^2^ Currently, the lineage tracing data show that more than 50 ​% of foam cells in atherosclerotic plaque are derived from VSMCs,^3^^,^^4^ but the mechanism of VSMC-derived foam cell formation remains unclear.

In fact, endothelial cells (ECs) in the lumen of blood vessel are subjected to a combined stimulation of longitudinal shear stress, circumferential stretch, and radial pressure, whereas VSMCs in the middle layer of the arterial wall are mainly subjected to stretch caused by pressure. Some in vitro studies have demonstrated that stretching can activate a wide range of cellular responses through various mechanoreceptors, such as integrins,^5^ ion channels,^6^ and piezo 1^6^^,^^7^. Oxidative stress is one of the most significant and prevalent cellular responses to mechanical stimuli. As a major pathway for cells to produce ROS, NADPH oxidases (NOX), especially NOX1, 2, and 4, have been frequently reported to be involved in various cellular responses of VSMCs, such as inflammation,^8^, ^9^, ^10^ proliferation,^11^^,^^12^ migration,^13^, ^14^, ^15^ and phenotypic transformation.^11^^,^^16^ For more than two decades, numerous studies have shown that activation of a variety of NOXs can induce oxidative stress and LDL oxidation.^8^^,^^12^^,^^13^^,^^17^, ^18^, ^19^, ^20^, ^21^, ^22^, ^23^, ^24^, ^25^ Some of them were used to explain the association between hypertension and atherosclerosis. Manea et al. reported that NOX1 and 4 in VSMCs can be regulated by NF-κB and JAK-STAT signaling pathways, revealing that NOXs are closely related to inflammatory response.^21^^,^^26^ Our previous data have also shown that 15 ​% stretching can cause a significant increase in IL-8 expression in both the single-type endothelial cell culture and multiple-type THP-1/EC/VSMC cell culture.^27^^,^^28^

Recently, Swiatlowska et al. investigated the effect of static high-pressure condition (180/120 ​mmHg) on lipid accumulation and transdifferentiation in VSMC using a homemade pressure chamber.^6^^,^^29^ Their results showed the static high-pressure condition induced calcium influx through Peizo1 located at the inner and outer membranes of the nucleus to trigger a range of mechanosignaling pathways, such as phospholipid and arachidonic acid signaling and epigenetic regulation. The pulsating blood pressure acting on the wall of the tube is partly converted into stretching, but their pressure device did not convert the static pressure into the stretching on cells. Our group once introduced a microfluidic device to simulate a three-dimensional curved stretch to induce the EC injury and inflammation.^28^ Based on this device, we also further developed a EC-VSMC double cell layer with the adherent THP-1 to induce the foam cell formation under 15 ​% stretch combined with 25 ​μg/ml LDL.^27^ However, we found that the lipid accumulation mostly occurred in the adherent THP-1 rather than VSMCs or ECs. This phenomenon may be due to the large ability of the macrophages derived from THP-1 to scavenge ox-LDL.

In general, the stretching of the blood vessel wall caused by blood flow pulsation is less than 7–8 ​%, and more than 10 ​% is considered to be beyond the normal range.^30^, ^31^, ^32^ Based on the susceptibility to atherosclerosis at the bend and bifurcation of blood vessels and the possibility of overstretching of the tube wall in case of blood retention, we speculate that there is a correlation between overstretching of the tube wall and atherosclerosis. Specifically, the formation of VSMC foam cells may be related to overstretching. To test this hypothesis, we used the same microfluidic stretch chips as our previous work and applied a stretch with a total area change rate of 15 ​% to the cultured VSMC alone for 24 ​h. The obtained results show that 15 ​% stretch causes a significant lipid accumulation in the VSMCs, along with a significant increase in NOX1 and CD36 protein expression. This stretching-induced lipid accumulation can be mitigated by NOX1 inhibitors, as well as antioxidants. Finally, the results also suggest that the increased NOX1 expression caused by 15 ​% stretching is associated with the proven JAK/STAT signaling pathway.

## 2 Results

### 2.1 15 ​% stretch causes lipid accumulation in VSMCs

**Fig. 1:**
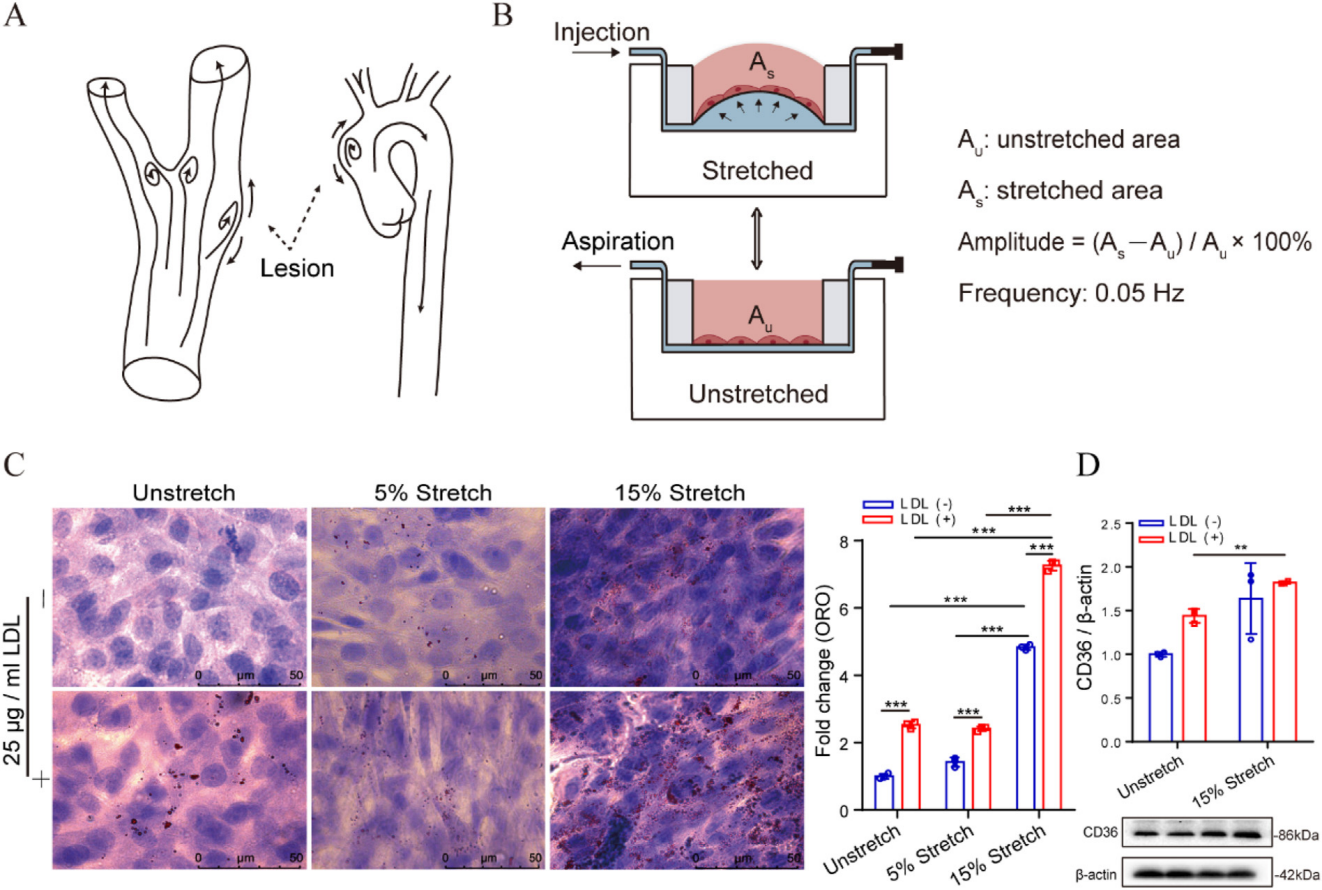
In vitro model of VSMC foam cell formation. (A) Diagram of vascular bulging image; (B) Working principle of the microfluidic chip; (C) Lipid accumulation in T/G HA-VSMCs assessed by Oil Red O staining; (D) Protein expression of CD36 in T/G HA-VSMCs. The data are shown as mean ​± ​SD (n ​= ​3), ∗*p* ​< ​0.05, ∗∗*p* ​< ​0.01, ∗∗∗*p* ​< ​0.001, ∗∗∗∗*p* ​< ​0.0001, and “ns” indicates no significance.

In Fig. 1A, a cartoon displays possible atherosclerosis-prone lesions at bend or bifurcation of blood vessels, where the wall of blood vessel may bulge outward due to disturbed blood flow.^33^ A home-made microfluidic device is used to provide non-uniform, multi-directional, and curved stretching to mimic the stretching profile at the protruding site of the arterial wall. Fig. 1B illustrates how the microfluidic device works, where the amplitude of stretching is the rate of change in the area at the bottom of each cell culture well under the stretched and unstretched conditions. As shown in Fig. 1C, the lipid accumulation labeled by Oil Red O (ORO) staining in VSMCs was only significantly increased at 15 ​% stretch and barely changed at 5 ​% stretch compared to no stretch condition. In addition, the added 25 ​μg/mL LDL to the medium significantly promoted lipid accumulation under all tested conditions. This may be due to the LDL containing some oxidized LDL or partial LDL being oxidized in situ by cell-generated ROS. At the same time, the change in the protein expression of CD36, a scavenger receptor of ox-LDL, is consistent with the corresponding lipid accumulation (Fig. 1D). This suggests that the expression of CD36 is sensitive to both the 15 ​% stretch and 25 ​μg/mL LDL. However, the expression of two cholesterol efflux transporters, ABCA1 and SR-BI, exhibit no significance between 15 ​% stretch and unstretch conditions (Fig. S1).

### 2.2 NOX1 is critical to stretch-induced lipid accumulation

**Fig. 2:**
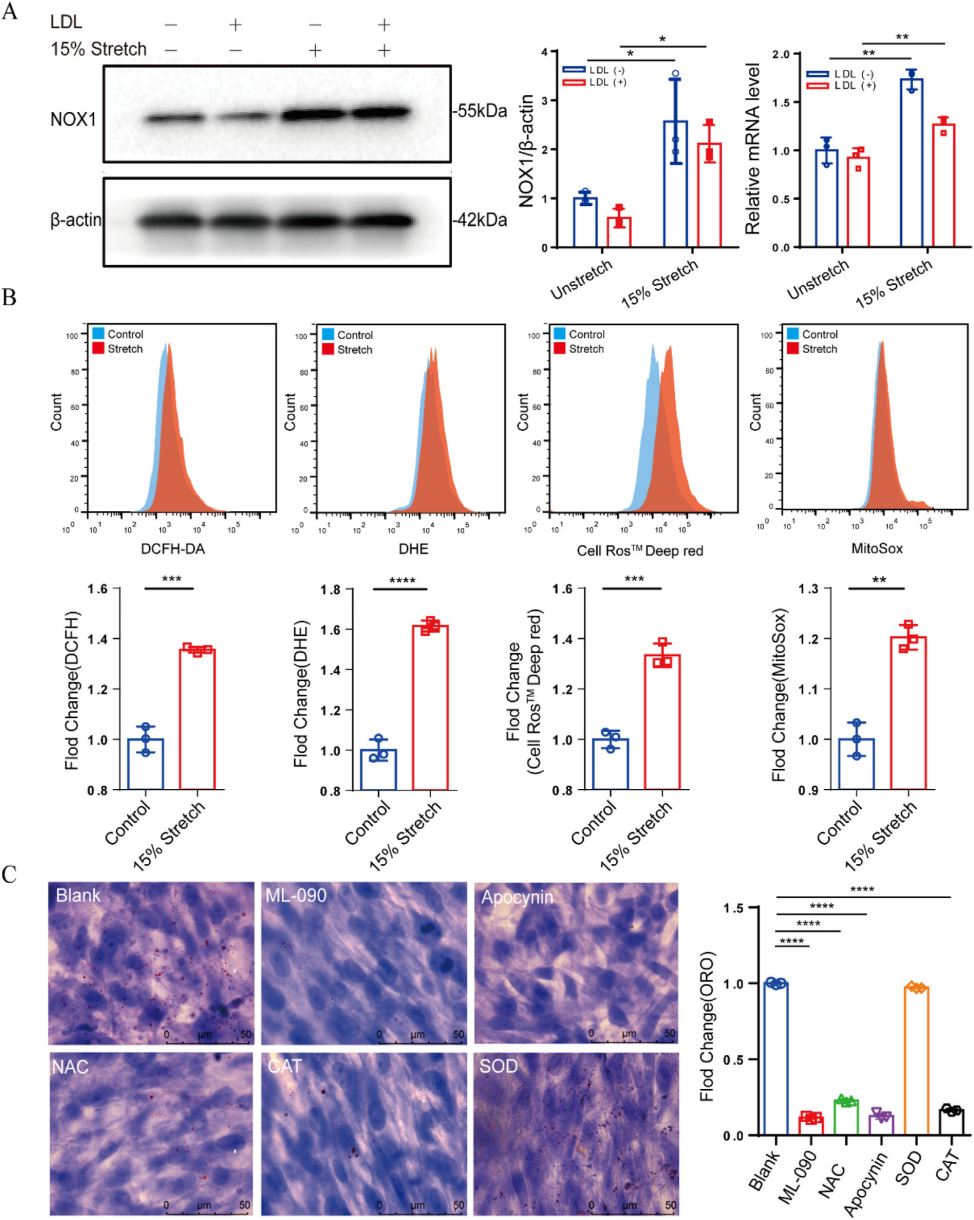
Roles of NOX1 and ROS in the stretch-induced lipid accumulation in VSMCs. (A) NOX1 transcription and protein expression; (B) ROS level indicated by DCFH, DHE, Deep Red and MitoSOX; (C) Stretch-induced lipid accumulation under different antioxidant treatment, ML-090 (10 ​μM), Apocynin (300 ​μM), NAC (1 ​mM), catalase (500 IU), SOD (100 IU). The data are shown as mean ​± ​SD (n ​= ​3), ∗*p* ​< ​0.05, ∗∗*p* ​< ​0.01, ∗∗∗*p* ​< ​0.001, ∗∗∗∗*p* ​< ​0.0001.

Ox-LDL is one of the key factors in the formation of foam cells and derived from the oxidation of extracellular LDL. It is important to identify the main sources of intracellular ROS for the understand of the lipid accumulation caused by stretching. Since NOXs is one of the main pathways for the production of ROS in cells, we aimed to compare the changes in transcription and protein levels of five NOXs. However, Fig. 2A and Fig. S2 show that 15 ​% stretching promotes more significant increase in both NOX1 transcription and protein expression, whereas other investigated NOXs lacked the consistency. Next, intracellular ROS levels were assessed using DCFH, DHE, Deep Red, and MitoSOX probes, and the results in Fig. 2B showed that 15 ​% stretching induced a significant increase in ROS levels indicated by these different probes, regardless of identifying as hydrogen peroxide, superoxide anion, or ROS in the cytoplasm and mitochondria. These elevated ROS levels should suggest that more extracellular LDL is oxidized. Furthermore, we selected ML-090, a NOX1-specific inhibitor, and Apocynin, a NOX-non-specific inhibitor, and three different ROS scavengers, N-acetyl cysteine (NAC), catalase, and superoxide dismutase (SOD), to validate the role of ROS elimination in the lipid accumulation. As shown in Fig. 2C, these reagents except for SOD have a significant inhibitory effect on the lipid accumulation. This suggests that the increase in ROS level associated with NOX1 plays an important role in the VSMC lipid accumulation. According to the SOD failure to reduce the lipid accumulation, we speculate that the extracellular LDL oxidation is mainly due to hydrogen peroxide rather than superoxide anion.

### 2.3 Knockdown of NOX1 alleviates stretch-induced lipid accumulation

**Fig. 3:**
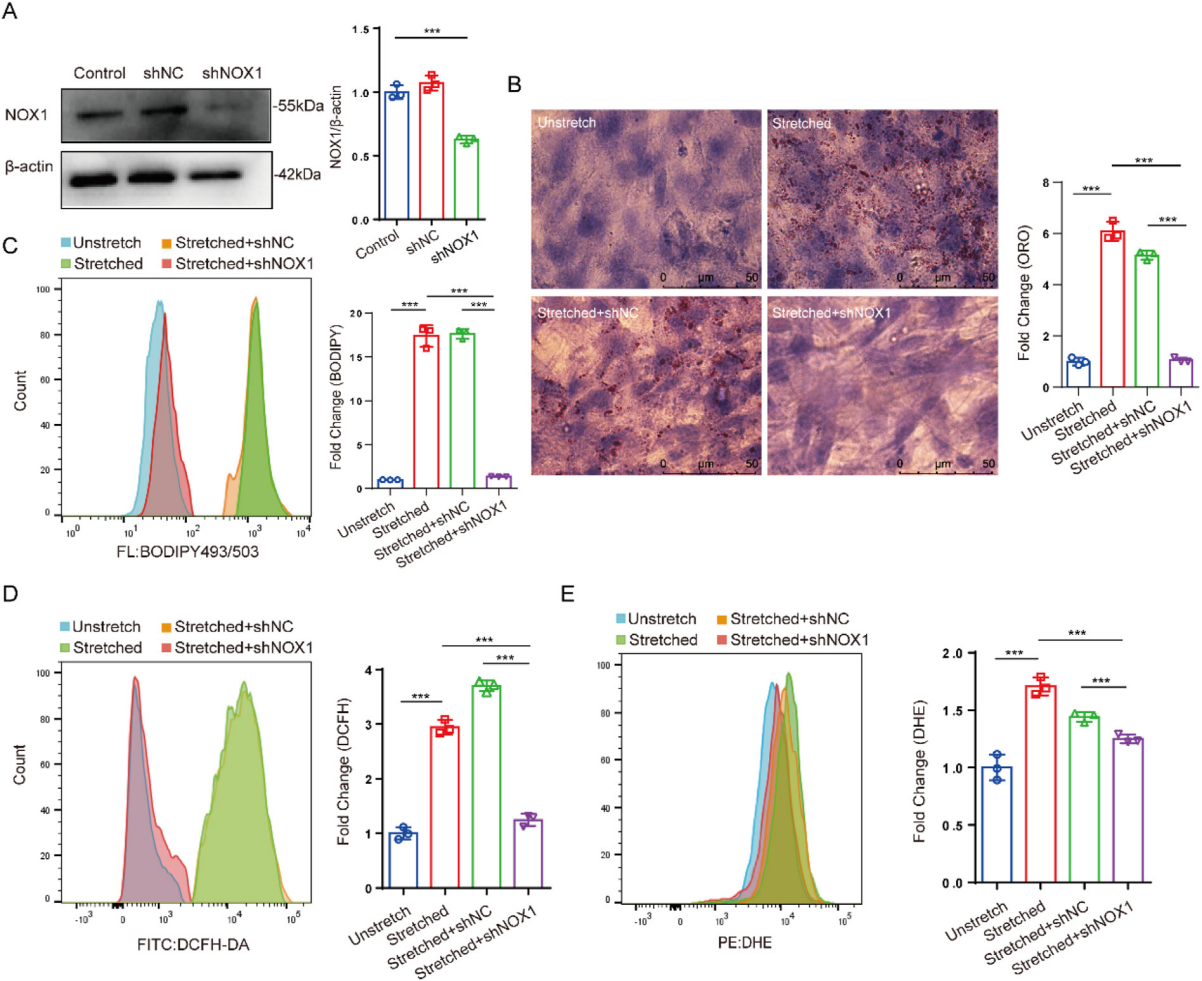
NOX1 knockdown reduces the stretch-induced lipid accumulation and ROS level in VSMCs. (A) Verification of NOX1 knockdown; (B and C) Lipid accumulation and lipid oxidation indicated by ORO and BODIPY, respectively; (D and E) ROS level indicated by DCFH and DHE. The data are shown as mean ​± ​SD (n ​= ​3), ∗p ​< ​0.05, ∗∗p ​< ​0.01, ∗∗∗p ​< ​0.001, ∗∗∗∗p ​< ​0.0001.

To evaluate the correlation between NOX1 expression and intracellular ROS level, NOX1 knockdown was performed by constructing a NOX1-deficient VSMC line using a lentiviral GFP vector (Fig. 3A). The NOX1 knockdown significantly reduced both the lipid accumulation and BODIPY-labeled intracellular lipid oxidation in the presence of 15 ​% stretch (Fig. 3B and C). The results of Fig. 3D and E show that NOX1 knockdown also significantly inhibited the stretch-induced ROS production indicated by either DCFH or DHE. However, the change in DCFH-labeled ROS level is much more pronounced than the DHE indicated change, suggesting that LDL oxidation is more dependent on hydrogen peroxide than superoxide anion. This is consistent with the results that SOD treatment did not alleviate lipid accumulation caused by stretching. As a result, these data demonstrate that NOX1 plays a key role in the stretch-induced lipid accumulation.

### 2.4 15 ​% stretch induces NOX1 upregulation via JAK/STAT signaling pathway

**Fig. 4:**
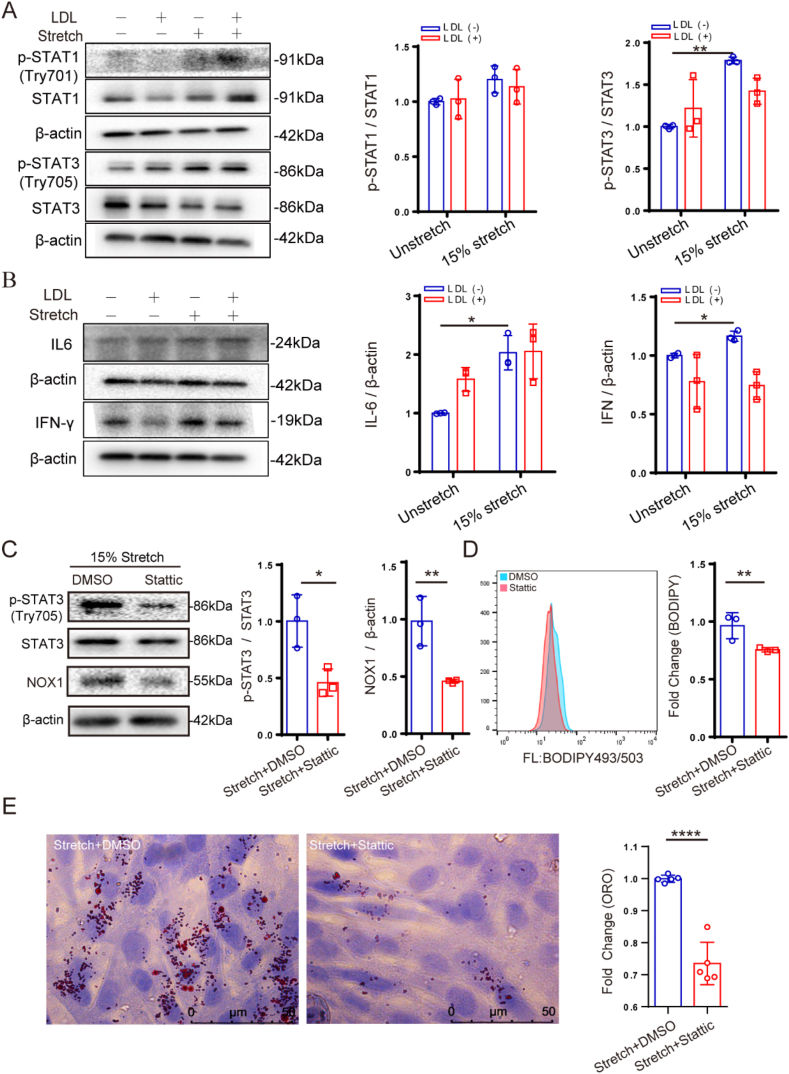
Stretch-induced lipid accumulation depends on the JAK/STAT signaling pathway. (A and B) 15 ​% stretch promotes the STAT1/3 phosphorylation and the expression of IL-6 and IFN-γ. (C–E) Inhibition of STAT3 phosphorylation and NOX1 expression, lipid oxidation, and lipid accumulation under 50 ​μM stattic treatment. The data are shown as mean ​± ​SD (n ​= ​3), ∗*p* ​< ​0.05, ∗∗*p* ​< ​0.01, ∗∗∗*p* ​< ​0.001, ∗∗∗∗*p* ​< ​0.0001.

It has been reported that the JAK/STAT signaling pathway regulates the expression of NOX1 in VSMC,^22^ so we examined several key factors of this signaling pathway. As shown in Fig. 4A and B, 15 ​% stretching induced the phosphorylation of STAT1 and STAT3, as well as the expression of IL-6 and IFN-γ. In contrast, the addition of LDL has no significant effect on this signaling pathway. On the other hand, we used stattic, a STAT3 phosphorylation inhibitor, to further confirm whether this pathway is involved in the regulation of NOX1 expression and lipid accumulation. As shown in Fig. 4C–E, stattic significantly inhibited NOX1 expression, lipid oxidation, and lipid accumulation. Therefore, the JAK/STAT signaling pathway can be targeted to inhibit NOX1 expression and lipid accumulation in VSMCs.

## 3 Discussion

Atherosclerotic plaque formation at arterial bends and bifurcations is closely associated with hemodynamic disturbances, where localized overstretching of the vascular wall may serve as a critical biomechanical trigger. While macrophage-derived foam cells have long been the focus of atherosclerosis research, emerging evidence highlights that VSMC contribute substantially to plaque foam cell populations.^2^^,^^3^ This study elucidates a novel mechanism by which pathological overstretch (15 ​%) directly induces lipid accumulation in VSMCs via NOX1-dependent oxidative stress, mediated through the JAK/STAT3 signaling pathway. These findings advance our understanding of VSMC-driven atherogenesis under biomechanical stress.

Our microfluidic model, which simulates non-uniform, multidirectional stretch mimicking vascular bulging, revealed that 15 ​% cyclic stretch significantly upregulated NOX1 expression and intracellular ROS levels in VSMCs (Fig. 2A and B). This ROS surge correlated with enhanced lipid accumulation, likely driven by the oxidation of native LDL in the microenvironment, as evidenced by the marked increase in CD36—a scavenger receptor for oxidized LDL (Fig. 1D). Notably, lipid accumulation was attenuated by NOX1 inhibition (ML-090), ROS scavengers (NAC, catalase), and NOX1 knockdown, underscoring NOX1 as a central mediator of stretch-induced oxidative stress (Fig. 2, Fig. 3). The inefficacy of SOD further suggests that hydrogen peroxide, rather than superoxide anion, predominantly mediates LDL oxidation in this context (Fig. 2C). These observations align with prior studies linking NOX1 to VSMC inflammation and phenotypic switching, yet uniquely identify its role in mechanotransduction-driven lipid accumulation. A key distinction of this work lies in the use of a VSMC monoculture system, which isolates the direct effects of stretch on VSMCs without confounding interactions with macrophages or endothelial cells. This approach revealed that dedifferentiated VSMCs, with inherently higher NOX1 and CD36 expression, are particularly susceptible to stretch-induced lipid accumulation. Such phenotypic plasticity underscores the heterogeneity of VSMCs in atherosclerosis and their capacity to adopt pro-atherogenic traits under mechanical stress. However, the reliance on a cell line rather than primary VSMCs warrants caution, as in vivo VSMCs may exhibit divergent responses influenced by their microenvironment. This perspective is supported by the results in Fig. S3, specifically, the lipid accumulatiorn and the expression of NOX1, CD36, and ABCA1 in primary VSMCs are significantly lower than those in the smooth muscle cell line.

Mechanistically, the JAK/STAT3 pathway emerged as a critical regulator of NOX1 expression under overstretch. Phosphorylation of STAT1/3 and elevated IL-6/IFN-γ levels indicate that mechanical stress activates inflammatory signaling, which in turn upregulates NOX1(Fig. 4A and B). The suppression of NOX1 and lipid accumulation by STAT3 inhibition (Stattic) solidifies this pathway's involvement(Fig. 4C). This aligns with reports that inflammatory cytokines like IFN-γ regulate NOX1 via JAK/STAT in VSMCs, but extends these findings to a biomechanical context. Importantly, our model highlights a feedforward loop: overstretch-induced endothelial damage may recruit inflammatory cells, releasing cytokines that amplify NOX1 expression in neighboring VSMCs, thereby exacerbating lipid retention and plaque progression.

Clinically, these findings underscore NOX1 as a potential therapeutic target to mitigate VSMC foam cell formation in high-risk vascular regions prone to overstretch. The efficacy of antioxidants and NOX1 inhibitors in our model supports further exploration of these agents in preclinical studies. Moreover, the identification of JAK/STAT3 as an upstream regulator opens avenues for repurposing existing anti-inflammatory therapies to disrupt mechanosensitive pathways in atherosclerosis.

## 4 Conclusions

In conclusion, this study establishes a direct link between pathological overstretch and VSMC foam cell formation, mediated via NOX1-driven oxidative stress and JAK/STAT3 activation. These insights not only deepen our understanding of biomechanical contributions to atherosclerosis but also highlight novel targets for intervention in vascular regions susceptible to hemodynamic stress.

## 5 Methods

### 5.1 Reagents and antibodies

Dulbecco's modified Eagle medium (DMEM) and fetal bovine serum (FBS) were obtained from Gibco (Thermo Fisher Scientific). Human LDL was obtained from Yiyuan Biotechnologies (Beijing, China). Oil red O powder was obtained from Biolabs (Beijing, China). Antibodies against NOX1 (1:2000, CSB-PA443876), NOX3 (1:2000, CSB-PA015960ESR2HU), and DUOX2 (1:2000, CSB-PA007229ESR1HU) were obtained from Cusabio (Wuhan, China). Antibodies against NOX2 (1:5000, ab129068), NOX4 (1:2000, ab109225), SR-B1 (1:1000, ab53629), ABCA1 (1:1000, ab18180) and IFN-γ (1:1000, ab133566) were from Abcam. Antibodies against CD36 (1:1000, 18836-1-AP), DUOX1 (1:2000, 67226-1-Ig) and IL-6 (1:600, 26404-1-AP) were from Proteintech. Antibodies against JAK2 (1:1000, 3230T), phospho-JAK2 (Tyr1007) (1:1000, 4406T), STAT1 (1:1000, 9172T), phospho-STAT1 (Tyr701) (1:1000, 7649T), STAT3 (1:1000, 9139T), phospho-STAT3 (Tyr705) (1:2000, 9145T), Stattic (97598), and protease/phosphatase inhibitor cocktail (100x, 5872S) were obtained from Cell Signaling Technology. Antibody against β-actin (1:10000, 109444-T32) was obtained from Sino Biological (Beijing, China). RNAprep Pure Micro Kit was obtained from Tiangen (Beijing, China). TransScript First-Strand cDNA Synthesis SuperMix was obtained from TransGen Biotech (Beijing, China). 2′, 7′-Dichlorodihydrofluorescein diacetate (DCFH-DA) was purchased from Sigma–Aldrich. Dihydroethidium (DHE) was purchased from ApexBio. Cell Ros™ Deep Red and MitoSOX™ Red Mitochondrial Superoxide Indicator was purchased from (Invitrogen). ML-090 (NOX1 inhibitor, 15172) and Apocynin (NOXs inhibitor, 119761) were obtained from Cayman Chemical (Ann Arbor, MI, USA). N-acetyl cysteine (NAC) and catalase was obtained from Yuanye Biotechnologies (Shanghai, China). Superoxide dismutase (SOD) was obtained from Beyotime (Shanghai, China).

### 5.2 Microfluidic stretching device

The microfluidic stretching device consists of three layers of polydimethylsiloxane (PDMS), an upper layer with array cell culture pores, a middle layer with stretchable deformation, and a lower layer with microfluidic fluid grooves. The working principle and stretch features are described in detail in our previous work.^27^^,^^28^ Briefly, one side of the microfluidic channel is blocked, while another side is connected to a syringe pump. After injecting a certain volume of water into the chamber just below the cell culture well, the middle layer membrane is deformed upwards to form a spherical cap, transducing stretch stimulus to attached cells. The amplitude is determined by the volume of injected water and the frequency is determined by the injection and aspiration duration. One cycle takes 20 ​s (0.05 ​Hz).

### 5.3 Cell culture

An aortic VSMC line (T/G HA-VSMC) is maintained by our laboratory and a human aortic smooth muscle cells (HUM-iCell-c010, primary) were purchased from Cell Technology Co., Ltd (Shanghai, China). Cells were cultured in DMEM containing 10 ​% FBS at 37 ​°C in a 5 ​% CO_2_ incubator. 50 ​μl of cell suspension (1 x 10^5^ cells/ml) was added into each well (6-mm diameter) coated with fibronectin. When the cells reached over 90 ​% confluence, the medium was replaced with fresh complete medium with or without the addition of 25 ​μg/ml LDL. Next, the syringe pump started to run with a program-controlled volume of liquid injection and aspiration, providing the desired stretch (15 ​%, 0.05 ​Hz), while the cells were cultured in non-stretched device as the control.

### 5.4 Oil Red O staining

After stretched for 24 ​h, the cells in each well were fixed with 4 ​% (m/v) paraformaldehyde in a phosphate-buffered saline (PBS) for 30 ​min, and then stained with 2.4 ​% (m/v) Oil Red O staining solution (Biolabs, Shanghai, China) for 1 ​h. The stained cells were incubated with 60 ​% (v/v) isopropanol solution for 15 ​s and followed by a hematoxylin solution (Biodragon Immunotechnologies, Beijing, China) for 3 ​min. Then, the hematoxylin solution was discarded and the cells were washed with tap water for 2–3 times. The cell-fixed membrane was cut off and mounted on a slide for observation. Images were acquired with a microscope (DM6B, Leica Microsystems) and the amount of the red lipid droplets was counted by Image J software (Wayne Rasband, NIH, USA).

### 5.5 Western blotting

After VSMCs in the wells were treated with the indicated conditions for 24 ​h, the cell lysates were prepared using RIPA buffer (Beyotime, Shanghai, China) with protease/phosphatase inhibitor phenylmethylsulfonul (PMSF), and the protein concentration was quantified using NanoDrop One (Thermo Scientific). The proteins were separated by 8.5 ​% SDS-PAGE, and then electrically transferred into a polyvinylidene difluoride (PVDF) membrane. Subsequently, the membranes were immersed in 5 ​% non-fat milk in Tris-buffered saline with Tween (TBST) at room temperature for 1 ​h with shaking. After that, the membranes were incubated with the indicated primary antibodies at 4 ​°C for overnight, following by the incubation of horseradish peroxidase (HRP)-labeled goat anti-rabbit or anti-mouse antibodies for 1 ​h at room temperature. The immunobands were visualized using an enhanced chemiluminescence (EMD, Millipore Corp., Burlington, MA, USA) and quantified by using an imaging system (Image Lab, Bio-Rad Laboratories).

### 5.6 qPCR

After treatment, the total RNA was isolated using RNAprep Pure Cell Kit (Tiangen, Beijing, China) according to the manufacturer's introduction. Single-strand cDNA was synthesized from 1 ​μg of total RNA using TransScript First-Strand cDNA Synthesis SuperMix (TransGen Biotech, Beijing, China). Quantitative analysis of NOX1-5, DUOX1, DUOX2, and GAPDH mRNA were carried out on Max3000P Real-time PCR instrument (Agilent, La Jolla, CA, USA) using TransStart Top Green qPCR SuperMix (+Dye II) (TransGen Biotech, Beijing, China). The primer sequences for target gene were as follows: *NOX1* (forward 5′-CTGCTTCCTGTGTGTCGCAA-3′ and reverse 5′-AGGCAGATCATATAGGCCACC-3′), *NOX2* (forward 5′-GTGCGTGCTGCTCAACAAGAGTT-3′ and reverse 5′-ATGGTGTGAATCGCAGAGTGAAGTG-3′), *NOX3* (forward 5′-CGGATTGTT CGAGGCCAAAC-3′ and reverse 5′-GCCAGAAAATTGAGGCACGG-3′), *NOX4* (forward 5′-AGCAGAGCCTCAGCATCTGTTCTT-3′ and reverse 5′-TGGTTCTCCTGCTTGGAACCTTCT-3′), *NOX5* (forward 5′-CTGCTGGTGCCTGGAATCTTGTT-3′ and reverse 5′-GCCGCTTGATGAGGAGATGAGTGA-3′), *DUOX1* (forward 5′-CGTGAGCACCCAAGCCTACAAAGT-3′ and reverse 5′-TCCCGCACATCTTCAACCAACACAT-3′), *DUOX2* (forward 5′-AGGATACCGTCCTTTCCTAGAC-3′ and reverse 5′-GGTTCTCCCGAATCCAGTAGTT-3′), *GAPDH* (forward 5′- ACATCAAGAAGGTGGTGAAGCAGGC-3′ and reverse 5′-GGTGTCGCTGTTGAAGTCAGAGGAG-3′). The mRNA level of each target gene was normalized to *GADPH* and analyzed using the 2^−ΔΔCt^ method.

### 5.7 ROS assays

After treated with the indicated conditions, the cells were washed twice with PBS. Subsequently, 50 ​μL of serum-free medium with 5 ​μM DCFH-DA, DHE, and Deep Red, MitoSOX was added into each cell culture well the incubation at 37 ​°C or 30 ​min. After that, the cells were collected in desired volume of PBS for flow cytometry (FACSAriaIII, Brand, USA) or microplate reader (BioTek, VT, USA) for measurement.

### 5.8 NOX1 knockdown

shRNAs for NOX1 constructs in lentiviral GFP vector (pLKO.1) were purchased from Tsingke Technologies (Beijing, China). In brief, HEK293T cells were transfected with either empty vector or target gene constructs with psPAX.2 and pMD2.G second-generation lentiviral packaging system. After 48 ​h incubation, the lentivirus particles in the medium were collected and filtered to infect the VSMCs. After 48 ​h infection, 2 ​μg/ml puromycin was added into the cell culture medium to obtain the stable cell lines with successful transduction. The following sequences, inserted into an empty pLKO.1-TRC vector, were used for short hairpin RNA (shRNA) knockdowns:

shScramble:

CCGGGAAGGCCTGAAGGTCAGATACCTCGAGGTATCTGACCTTCAGGCCTTCTTTTTT.

NOX1-shRNA1:

CCGGGCCTATATGATCTGCCTACATCTCGAGATGTAGGCAGATCATATAGGCTTTTTT.

NOX1-shRNA2:

CCGGCCGCACACTGAGAAAGCAATTCTCGAGAATTGCTTTCTCAGTGTGCGGTTTTTT.

NOX1-shRNA3:

CCGGCCAAGGTTGTTATGCACCCATCTCGAGATGGGTGCATAACAACCTTGGTTTTTT.

NOX1-shRNA4:

CCGGGAACAGGAGATGGAGGAATTACTCGAGTAATTCCTCCATCTCCTGTTCTTTTTT.

### 5.9 BODIPY staining

The cells in the each well were rinsed with PBS three times and incubated in 2 ​μM 4,4-difluoro-1,3,5,7,8-pentamethyl-4-bora-3a,4a-diaza-s-indacene (BODIPY 493/503, Cayman Chemical) staining solution in the dark for 15 ​min at 37 ​°C, following by washing with PBS. The cells were trypsinized and centrifuged with 250 ​g for 5 ​min at 4 ​°C. After that, the supernatant was aspirated carefully and the cells were resuspended in 500 ​μl PBS. Flow cytometry was used for quantification at 488 ​nm/525 ​nm.

### 5.10 Statistical analysis

The data are expressed as the means ​± ​SD. Comparisons between two groups were performed using the unpaired Student's t-test, and comparisons among three or more groups were performed using one-way or two-way analysis of variance (ANOVA) followed by Bonferroni's multiple comparison test. P values ​< ​0.05 were considered statistically significant. All statistical analyses were performed with GraphPad Prism 6.01.

## CRediT authorship contribution statement

**Jiazhen Zhang:** Methodology, Formal analysis, Data curation. **Qinfen Li:** Methodology, Investigation, Formal analysis, Data curation. **Suoqi Ding:** Methodology, Investigation. **Wei Xu:** Methodology, Investigation. **Jilei Su:** Visualization. **Jingang Cui:** Visualization. **Yongsheng Ding:** Writing – review & editing, Writing – original draft.

## Data availability

The datasets used and/or analysed during the current study available from the corresponding author on reasonable request.

## Ethical approval

This study does not contain any studies with human or animal subjects performed by any of the authors.

## Declaration of competing interest

The authors declare that there are no financial interests/personal relationships that could be considered as potential competing interests that would affect the work reported in this paper.
