# Supplementary material for: Overstretch causes lipid accumulation in vascular smooth muscle cells dependent on NADPH oxidase 1
Source: Mechanobiol Med. 2025 Mar 26;3(2):100129. doi: 10.1016/j.mbm.2025.100129 (PMC12067898; doi:10.1016/j.mbm.2025.100129)
Supplement: Multimedia component 1 [file mmc1.docx]

**Supplementary information**

**Overstretch causes lipid accumulation in vascular smooth muscle cells dependent on NADPH oxidase 1**

Jiazhen Zhang^1^, Qinfen Li^1^, Suoqi Ding^2^, Wei Xu^1^, Jilei Su^1^, Jingang Cui^1^, Yongsheng Ding^1*^

^1^College of Life Sciences, University of Chinese Academy of Sciences, Beijing 101408, China

^2^School of Chemistry, Beihang University, Beijing, 100191, China

*Corresponding author: Yongsheng Ding

Email: [dingysh@ucas.edu.cn](mailto:dingysh@ucas.edu.cn)


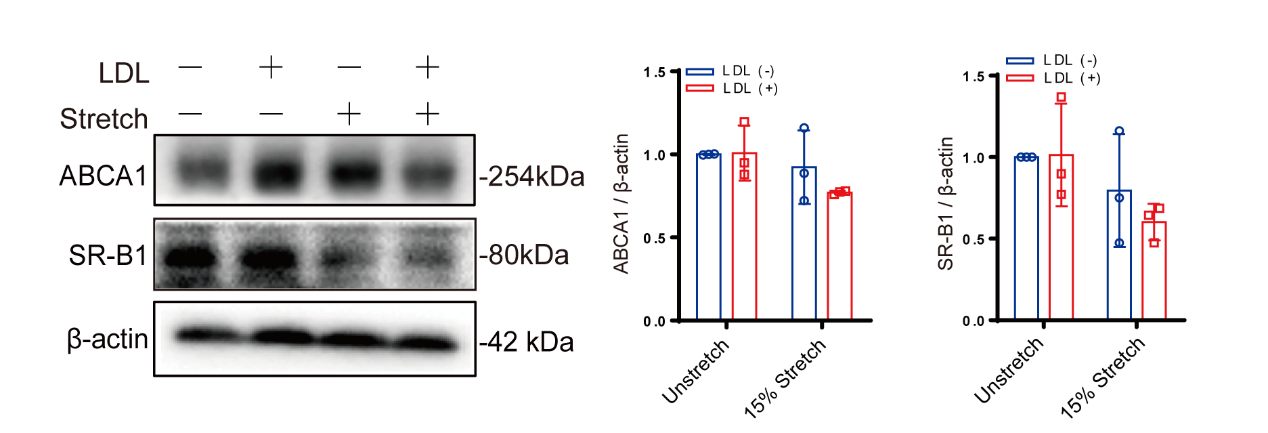


Figure S1. Protein expression of ABCA1 and SR-B1 in T/G HA-VSMCs under the combination of 15% stretch and 25µg/ml LDL. The data are shown as mean ± SD (n = 3), *p < 0.05, **p < 0.01, ***p < 0.001, ****p < 0.0001, and “ns” indicates no significance.


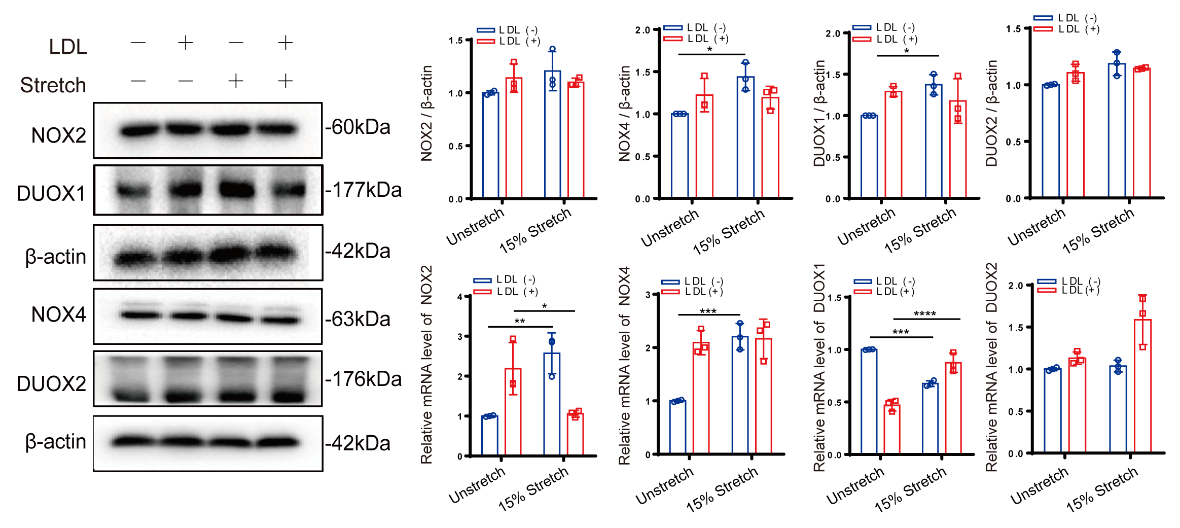


Figure S2. Transcriptional and protein expression of other four NOXs in T/G HA-VSMCs under the combination of 15% stretch and 25µg/ml LDL. The data are shown as mean ± SD (n = 3), *p < 0.05, **p < 0.01, ***p < 0.001, ****p < 0.0001, and “ns” indicates no significance.


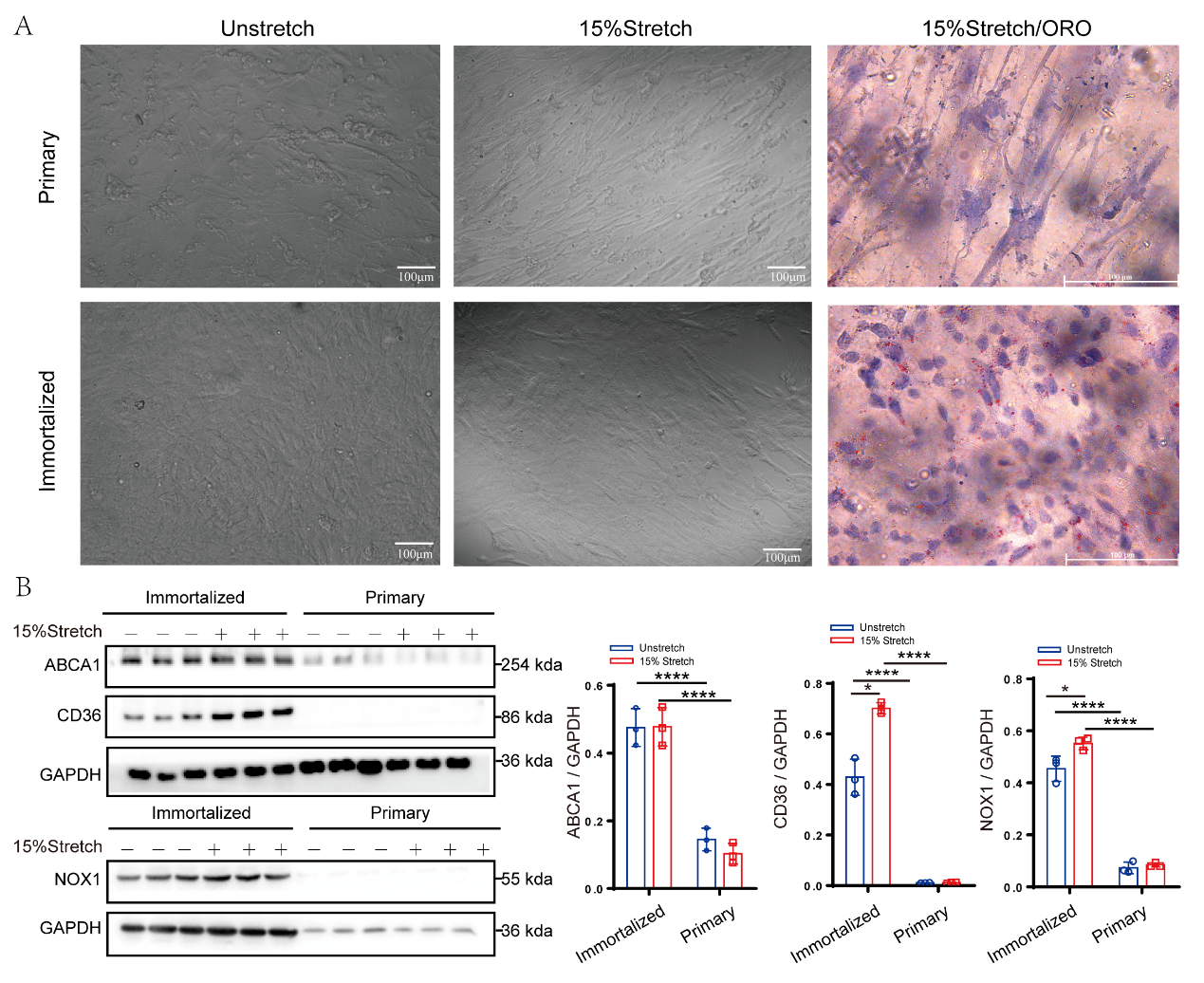


Figure S3. Comparison of morphological, ORO staining, and WB results between an aortic VSMC line (T/G HA-VSMC) and a human aortic smooth muscle cells (HUM-iCell-c010, primary). The data are shown as mean ± SD (n = 3), *p < 0.05, **p < 0.01, ***p < 0.001, ****p < 0.0001, and “ns” indicates no significance.
